# Relationship between maternal grit and effortful control among 18–21-month-old toddlers

**DOI:** 10.3389/fpsyg.2024.1346428

**Published:** 2024-05-17

**Authors:** Awoun Jung, Mikako Ishibashi, Yuta Shinya, Shoji Itakura

**Affiliations:** ^1^Department of Psychology, Ochanomizu University, Tokyo, Japan; ^2^Graduate School of Education, The University of Tokyo, Tokyo, Japan; ^3^Department of Psychology and Humanities, College of Sociology, Edogawa University, Chiba, Japan; ^4^Research Organization of Open Innovation and Collaboration Ritsumeikan University, Osaka, Japan

**Keywords:** grit, parenting, toddler, effortful control, self control

## Abstract

Grit is known to be effective for long-term academic and social success. However, few studies have focused on the role of grit in parenting and its effect on the development of grit in children. Therefore, this study investigated the effect of maternal grit on children’s effortful control (EC), which is thought to be a precursor to grit, using parenting as a mediating factor. Participants in the current study were 412 children (age range: 18–21 months, *M* = 34.67 months, *SD* = 4.51 months) and their mothers. We assessed maternal grit, parenting style, maternal EC, and child EC, and found that maternal grit, maternal EC, and parenting style were positively correlated with child EC. Furthermore, maternal grit was related to EC in children not only directly, but also indirectly through responsive parenting. Additionally, maternal grit was found to be directly related to child EC only when assessed separately from maternal EC. The current study’s findings suggest that maternal grit is directly related to EC in children in a way that differs from the mother’s EC in child-rearing situations.

## 1 Introduction

Grit, which has recently attracted interest, has been defined as “perseverance and passion for long-term goals,” which entails maintaining effort and interest in long-term goals despite adversity (Duckworth et al., 2007). The grit scale was developed by Duckworth et al. (2007) as a two-factor self-report scale for describing and predicting long-term outcomes based on perseverance of effort and consistency of interest in long-term goals. They also showed that people with higher grit scores have higher achievement outcomes, such as academic success and spelling contests. Extant research has focused on the effects of individual grit on one’s own abilities, such as academic performance and socioeconomic status (SES), with few studies conducted on the effects of personal grit on other factors, especially parenting (Oriol et al., 2017; Alhadabi and Karpinski, 2020; Li and Li, 2021). For example, parenting may be related to grit in that parents continue to maintain effective support and attention toward long-term goals for their own child.

However, little is known about the role of grit in parenting, or its effects on the development of grit in children. In an experimental setting, 12–15 months infants who observed adults effortfully engaging in a task engaged in tasks for a longer duration than those who observed adults engaging in a task with less effort (Leonard et al., 2017). Similarly, Shinya and Ishibashi (2022) revealed that observing adults’ effortful behavior affected infants’ enhanced sustained goal-directed attention across contexts. A recent study reported that maternal grit was positively associated with maternal responsiveness, controlled parenting, and grit among children aged 3–6 years (Imafuku et al., 2021). Additionally, maternal parenting style was positively associated with maternal perseverance of effort, suggesting that children raised by parents with high grit learn about persistence through parenting. These findings suggest that social engagement by caregivers with persistence may influence the development of effortful behavior in infants, although the long-term mediating effect of parenting on grit in children has not yet been directly investigated.

Most research on grit has been conducted on adolescents and older individuals (Oriol et al., 2017; Kevenaar et al., 2023), while few studies have focused on grit in children (Imafuku et al., 2021), as there are no standardized methods of measuring grit and the formation process of grit remains subject to debate. Furthermore, a critical issue in fostering grit in children is determining how parental grit relates to the precursors of grit at earlier developmental stages, when grit in children has not yet been identified (Lucca and Sommerville, 2018). One candidate precursor of grit is self-control, which is often referred to as a concept similar to grit. Self-control is derived from social psychology and involves the use of cognitive processes to regulate behavior, focus or shift attention, and inhibit behavior to achieve subdominant goals (Yamagata et al., 2005a; Karreman et al., 2006; Nigg, 2017). In the context of developmental psychology, self-control is often referred to as effortful control (EC), which is the ability to inhibit a dominant response and activate a subdominant response (Ahadi and Rothbart, 1994; Rothbart and Bates, 2006).

Duckworth and Gross (2014) argued that self-control is the ability to regulate or resist attention in the presence of temptation, whereas grit involves consistently pursuing a higher-order goal over years or sometimes decades. Grit, self-control, and EC are indistinguishable at both the phenotypic and behavioral genetic levels of conscientiousness, suggesting that the overlap is substantially due to genetic factors (Takahashi et al., 2021). Similarly, grit and self-control are significantly phenotypically correlated with academic performance, suggesting that these variables may share genetic factors (Kevenaar et al., 2023). By contrast, the relationship between grit and EC still requires discussion, and some argue that grit and EC are different concepts. Studies on elementary and junior high school students have shown differences between grit and self-control in the context of academic performance (Oriol et al., 2017). By estimating the covariance of both components, grit was found to be associated with academic self-efficacy at both educational stages, but only with school satisfaction at secondary school. Conversely, self-control showed a significant relationship with school satisfaction only among elementary school students. Taken together, these findings suggest that while grit and self-control share overlapping components, they may represent distinct concepts with unique components, especially in terms of the timescale associated with goal attainment.

Nevertheless, in children at an early stage of development, it is difficult to maintain long-term goals because episodic memory formation and mental time travel are underdeveloped (Suddendorf and Redshaw, 2013; Blankenship and Kibbe, 2019) Therefore, it is possible that “EC (self- control),” which controls behavior for shorter-term goals, precedes and underlies “grit,” which is the ability to persistently pursue long-term goals (Lucca and Sommerville, 2018). Actually, early EC is thought to emerge in the first year of life (Diamond, 1991, 2002), and has been shown to a predictor for later self-control and academic outcomes (Blair and Razza, 2007; Valiente et al., 2010; Neuenschwander et al., 2012). Therefore, the present study focused on the EC—a representative measure of self-control and persistence in developmental psychology—as a child outcome predicted by maternal grit.

Moreover, assuming that grit and EC represent different variables, it is conceivable that the influence of maternal grit and EC on child EC differs when mediated by parenting. Lengua et al. (2007) reveal that not all aspects of parenting are associated with EF (which often overlaps with EC). It has been suggested that parents with high EF skills may refrain from indulgent and over-reactive, but positive parenting such as engaging children in family-discussions and encouraging them persistently, may not necessarily depend on high EF (Korucu et al., 2019). Thus, maternal EC and maternal grit may therefore differ in their impact on parenting. Parents should demonstrate “self-control” by selecting certain behaviors, such as an appropriate response to their child, while inhibiting other behaviors, such as yelling. Further, parents must demonstrate “grit,” which persistently focuses on attaining long-term goals and sustaining their passion, to their children. Although there is some evidence that observing others’ effortful behavior over a short period in a general non-parenting context encourages children to imitate and encourage effortful behavior (Leonard et al., 2017; Shinya and Ishibashi, 2022), the long-term effects of observing caregivers’ effortful behavior are still unknown. Therefore, the present study examined the possibility of different effects of parental grit and parental EC in the context of parenting.

Individual differences in child EC arise from both genetics and environmental factors (Losoya et al., 1997; Willems et al., 2019). Early developmental stage of EC is heavily influenced by parents (e.g., Cumberland-Li et al., 2003; Eisenberg et al., 2005; Davenport et al., 2011); warm and supportive parenting not only contributes to predicting future of child EC (Kochanska et al., 2000; Eisenberg et al., 2005), but also improves cognitive solving skills during childhood (Whipple et al., 2011). Additionally, parenting styles have been found to be affected by parents’ EC. For example, mothers with high EC were found to exhibit higher levels of warmth, support, and positive emotions towards their children (Mangelsdorf et al., 1990; Cumberland-Li et al., 2003; Davenport et al., 2011). Although parental EC and parenting influence child EC, research on the influence of parental grit remains scarce. Imafuku et al. (2021) examined child EC, parent–child grit, and parenting, and observed a positive correlation between each variable; however, the direct effect of parental grit on child EC and the indirect effect of parenting were not examined.

This study aimed to clarify the effects of maternal grit, maternal EC, and parenting style as strong predictors of child EC. To measure child EC, we conducted a questionnaire survey with mothers of children aged 18–21 months to determine the earliest stage of child EC (using the Early Child Behavior Questionnaire [ECBQ]; Putnam et al., 2006). As children’s EC become more consistent around the age of 3–4 years (Kochanska et al., 2000), examining the children’ EC before 2 years of age, which is not crystalized the EC, is considered appropriate to examine the precursors of grit in children. We hypothesized that maternal grit would positively correlate with child EC and parenting (Imafuku et al., 2021). In addition, previous studies have shown that maternal grit is associated with responsive parenting (Imafuku et al., 2021) and that warm parenting promotes EC in children (Kochanska et al., 2000; Eisenberg et al., 2005). Thus, we hypothesized that maternal grit predicts EC in children mediated by parenting style. Finally, given that structural differences between grit and EC have been debated (Duckworth et al., 2007; Duckworth and Gross, 2014), we explored whether maternal grit is associated with child EC through parenting in the same way as parental EC. Accordingly, we hypothesized that maternal grit is a strong predictor of child EC, even after controlling for parenting.

## 2 Materials and methods

### 2.1 Participants

This study was approved by the ethical committee of Life Science Research Ethics and Safety, The University of Tokyo (approval number: 17–270). The participants were primary caregivers of children aged 18–21 months. They were recruited from a database using an online survey (Cross Marketing, Inc., Tokyo, Japan). We conducted the survey twice, obtaining 714 responses from March 22 to 24, 2021, and 644 responses from October 22 to November 1, 2021. To ensure the validity of responses, trap questions randomly inserted within the survey items were excluded. Additionally, all straight-line responses were excluded, resulting in the exclusion of 526 out of 1,358 respondents. Furthermore, responses with significantly long durations (nearly an hour or more) were excluded based on recorded start and end times. Of the collected data, 138 sets were from fathers; however, these were excluded from the current analysis for use in other studies. Hence, we utilized 412 data from mothers only. After obtaining all responses, we excluded the following data: respondents (1) who were not primary caregivers, including fathers, (2) whose children were outside the 37–45 week gestation range, and (3) who provided contradictory and incomplete answers. The final sample included 412 mothers (mean age = 34.67 months, *SD* = 4.51 months) of children aged 18–21 months (202 girls [49%], 210 boys [51%], mean age = 19.78 months, *SD* = 1.02 months). The mothers’ educational levels ranged from middle school to doctoral/postgraduate degrees as follows: middle school (2.2% of participants), high school (17.5%), college (32%), bachelor’s degree (42%), master’s or doctoral degree (5.1%), and other academic backgrounds (1%). The mothers self-reported family income (in Japanese yen) across 12 categories was as follows: 0–1,000,000 (1.2% of participants); 1,000,001–2,000,000 (2.9%); 2,000,001–3,000,000 (6.3%); 3,000,001–5,000,000 (28.6%); 5,000,001–6,000,000 (16%); 6,000,001–7,000,000 (14.3%); 7,000,001–9,000,000 (14.3%); 9,000,001–10,000,000 (4.4%); 10,000,001-12,000,000 (5.3%); 12,000,001–15,000,000 (3.4%); 15,000,001-20,000,000 (1.9%) and > 20,000,001 (1.2%).

### 2.2 Measurements

The survey comprised five sections: (1) Japanese version of the Short Grit Scale (Grit-S; Takehashi et al., 2018), (2) parenting style (Nakamichi, 2013), (3) parenting EC (Yamagata et al., 2005b), (4) ECBQ-Short Form (Nakagawa et al., 2011), and (5) demographic information.

#### 2.2.1 Maternal grit

Grit was assessed using Japanese version of Short Grit Scale (Grit-S, Takehashi et al., 2018), which comprises 12 items that assess two factors: six items for perseverance of effort (e.g., I have overcome setbacks to conquer an important challenge) and six items for consistency of interest (e.g., new ideas and projects sometimes distract me from previous ones). Each item is rated on a five-point Likert scale ranging from 1 (not at all like me) to 5 (very much like me). The overall grit score was calculated as the average of total scores of the two factors. The internal consistency of the scales demonstrated acceptable reliability (effort: *α* = 0.86; interest: *α* = 0.77). The entire scale demonstrated acceptable internal consistency (*α* = 0.75).

#### 2.2.2 Parenting style

Parenting style was assessed using the Japanese version of the Parenting Style Questionnaire (Nakamichi, 2013), which was based on Baumrind (1966). The questionnaire comprises 13 items that assess two factors: eight items for responsiveness (e.g., expressing affection by hugging and talking with gentle words) and five items for control (e.g., keeping your child quiet in places that require them to be quiet, such as libraries and movie theaters). Items are scored using a four-point Likert scale ranging from 1 (never) to 4 (always). The total responsiveness score ranges from 8 to 32, and that of control ranges from 5 to 20. The scores on the two dimensions of parenting (parenting score) were averaged for each sub-factor. Internal consistency values were *α* = 0.84 for responsive parenting and *α* = 0.71 for controlling parenting. The two subscales were significantly correlated (*r* = 0.47, *p* < 0.001) and a composite score was created by calculating the average of the total scores of both subscales.

#### 2.2.3 Maternal EC

Maternal EC was measured using the Japanese version of Adults Temperament Questionnaire (ATQ; Rothbart et al., 2000) developed by Yamagata et al. (2005b). The ATQ includes four factors: negative affect, extraversion/surgency, EC, and orienting sensitivity. In this study, we used the 35 items in the EC dimension of ATQ, based on previous studies (Tanaka et al., 2013; Nishimura and Matsuda, 2020). This dimension comprises three components: (1) inhibitory control—the ability to inhibit inappropriate behaviors (11 items); (2) activation control—the ability to perform an action in a situation that one has a strong tendency to avoid (12 items); and (3) attentional control—the ability to focus and shift attention (12 items; Rothbart et al., 2000). Each item is scored on a five-point Likert scale ranging from 1 (not true) to 5 (true). The score ranges from 11 to 55 for inhibitory control and from 12 to 60 for attentional control and activation control. Total maternal EC score was the average of the entire scale. Items in each of the subscales demonstrated adequate internal consistency (inhibitory control: *α* = 0.65; activation control: *α* = 0.79; and attentional control: *α* = 0.82). The 35-item EC scale demonstrated acceptable reliability in our sample (*α* = 0.89).

#### 2.2.4 Child EC

We measured child EC using Japanese version Early Childhood Behavior Questionnaire-short form (ECBQ; Nakagawa et al., 2011), comprising 107 items that assess 18 dimensions of temperament in children aged 18–36 months. We used only 34 items related to EC; based on previous studies (Nakagawa et al., 2011; Inoue and Kondo, 2019), EC is a validated factor defined by the loadings of perceptual sensitivity (PS), attention shifting (AS), sociability (SC), positive anticipation (PA), low-intensity pleasure (LIP), and attention focusing (AF). Each item was scored on a seven-point Likert scale ranging from 1 (never) to 7 (always), and an 8 option of “not applicable.” The total score of child EC was calculated as the average total score of all the sub-factors. The internal consistency each sub-factor was as follows: PS (*α* = 0.78), AS (*α* = 0.70), SC (*α* = 0.96), PA (*α* = 0.88), LIP (*α* = 0.84), and AF (*α* = 0.55). The entire scale demonstrated acceptable reliability (*α* = 0.94). We excluded 1% of the data that were more than three standard deviations above or below the mean of the total number of eight points (not applicable).

### 2.3 Data analysis

The demographic information included the child’s sex and age, maternal age, maternal academic background, and family income. Before conducting the full-scale analysis, SES was standardized. SES was assessed based on family income and maternal education. Family income was assigned a 12-point scale and maternal education was assigned a 5-point scale. Following a previous study (Moriguchi and Shinohara, 2019), family income and maternal education were individually converted to z-scores and averaged to create a total SES score.

To assess the first hypothesis we calculated descriptive statistics and correlations between the variables. Considering the second research question, a multiple regression model was used to examine the potential predictive relationships among maternal grit, maternal EC, parenting score, and child EC. To investigate whether maternal grit is associated with child EC, we conducted mediation analyses using AMOS (ver. 28). Finally, we examined the mediating role of parenting in the relationships between maternal grit, maternal EC, and child EC. We tested the significance of the indirect effect using bias-corrected bootstrapping procedures with 2,000 simulations. A bias-corrected bootstrapped effect with a 95% confidence interval (CI) that does not include zero would be evidence of an intermediary pathway linking maternal grit and maternal EC to child EC through the two dimensions of parenting.

## 3 Results

### 3.1 Correlation analysis

Table 1 summarizes the means and standard deviations for the study variables. A series of correlation analyses were used to examine the relationships between the variables. Maternal grit was positively associated with responsiveness parenting (*r* = 0.18, *p* < 0.001), maternal EC (*r* = 0.51, *p* < 0.001), and child EC (*r* = 0.24, *p* < 0.001). Further, child EC was positively associated with responsive parenting (*r* = 0.33, *p* < 0.001), controlled parenting (*r* = 0.24, *p* < 0.001), and parental EC (*r* = 0.28, *p* < 0.001). The correlations between maternal EC and responsive parenting (*r* = 0.31, *p* < 0.001) and controlling parenting (*r* = 0.16, *p* < 0.01) were positive and statistically significant. Only maternal EC was positively associated with children’s age (*r* = 0.12, *p* < 0.05). Children’s sex was also positively associated with only controlled parenting (*r* = 0.10, *p* < 0.05). SES was not correlated with any of the other variables.

**Table 1:** Means, standard deviations, and correlations among the study variables.

	*M*	*SD*	1	2	3	4	5	6	7	8
1. Age (in months)	19.78	1.02	—							
2. Sex	—	—	0.1	—						
3. SES	−0.05	0.76	0.05	−0.03	—					
4. Responsiveness parenting	3.24	0.43	0.04	0.01	0.01	—				
5. Controlled parenting	3.11	0.48	−0.07	0.10*	0.01	0.47***	—			
6. Maternal Grit	3.02	0.49	0.02	−0.02	0.06	0.18***	0.05	—		
7. Maternal EC	2.72	0.36	0.12*	0.01	0.08	0.31***	0.16**	0.51***	—	
8. Child EC	4.69	0.98	0.06	−0.01	−0.02	0.33***	0.24***	0.24***	0.28***	—

Standardized effects were reported. Maternal grit, Grit short scale; Responsiveness parenting, Controlled parenting, Parenting style questionnaire; Child EC, early childhood behavior questionnaire short form. *p < 0.05, **p < 0.01, ***p < 0.001.

### 3.2 First mediation model

**Figure 1:**
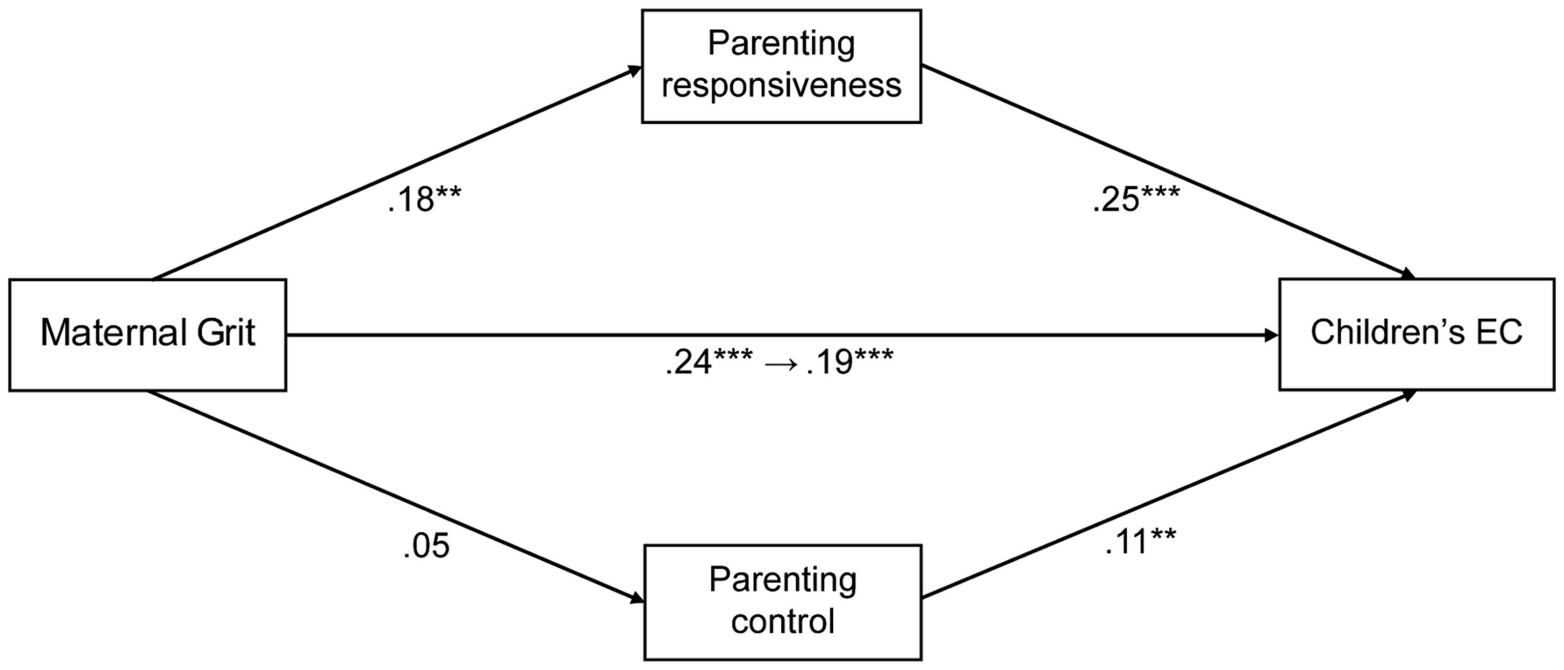
Mediation model of maternal grit on children’s effortful control. Demographic covariates not shown. Standardized effects were reported. The number before the arrow from “Maternal grit” to “Child EC” is the value before introducing the parameter, and the number after is the value after introducing the parameter. *p < 0.05; **p < 0.01; ***p < 0.001.

A structural equation model was used to examine parenting as a mediator between maternal grit and child EC. We used a mediation model via AMOS. The results (see Figure 1) revealed that the total standardized direct effect of maternal grit on child EC, without considering the effect of parenting, was significant (*β* = 0.24, *p* < 0.001). The analysis of the association between maternal grit and responsive parenting suggested a significant positive effect (*β* = 0.18, *p* < 0.01), while the coefficient of controlled parenting was not significant (*p* > 0.05). Independently, both responsive and controlled parenting significantly predicted child EC (*β* = 0.25, *p* < 0.001; *β* = 0.11, *p* < 0.05). Finally, the regression model indicated that the standardized direct effect of maternal grit on child EC, considering the effect of parenting, was significant (*β* = 0.19, *p* < 0.001), but the effect was smaller than before the mediation variable was considered.

Although we evaluated the fit of this model, it did not show a high degree of fit (*χ*^2^/*df* = 99.604, *p* = 0.00, CFI = 0.443, RMSEA = 0.490). Because the number of observed variables and sample size tend to produce estimates that suggest a poorer fit than their population counterparts in CFI and RMSEA (Shi et al., 2019), it is difficult to conclude that this model is not necessarily appropriate. Furthermore, it is recommended to supplement reliability by using bootstrapping methods in mediation analyzes for small to medium-sized samples (Shrout and Bolger, 2002). Therefore, to ascertain the significance of the effects, a bootstrap nonparametric resampling procedure with 2,000 bootstrap sample simulations with bias-corrected 95% confidence intervals (CI) was applied. Bootstrap analysis of the indirect effect of parenting on the association between maternal grit and child EC showed a bias-corrected 95% CI that did not include zero (CI [0.01, 0.10]).

These results, along with the statistical significance of the remaining paths, support the idea of a partial mediation model that accounts for 14.7% of the variance in child EC (*F* [3,408] = 24.612, *p* < 0.001). Children’s age and sex, SES were not statistically significant associated with child EC and were removed from subsequent mediation analysis in SEM.

### 3.3 Second mediation model

Overall, the model that additionally considered maternal EC to the first mediation model accounted for 15.8% of the variance in child EC (*F* [4,407] = 20.232; *p* < 0.001). The results from the mediation model suggest that the indirect effect of parenting on the association between maternal grit and child EC disappeared, and only a partial mediation between maternal and child EC was observed. More specifically, with both parenting dimensions as mediators, the estimated regression coefficient between maternal grit and parenting score was reduced to the point that maternal grit was no longer a significant predictor (*β* = 0.04, *p* = 0.494; *β* = −0.04, *p* = 0.499). Additionally, the standardized direct effect of maternal grit on child EC was lower than that in the first model (*β* = 0.13, *p* < 0.05).

By contrast, the estimated regression coefficient between maternal EC and responsive parenting was positive and significant (*β* = 0.29, *p* < 0.001). Additionally, the coefficient of controlled parenting was positive and significant (*β* = 0.18, *p* < 0.01). Moreover, the standardized direct effect of maternal EC on child EC, considering the effect of parenting, was significant (*β* = 0.14, *p* < 0.05). Finally, the analysis of the association between both parenting dimensions and child EC suggested a significant positive effect (*β* = 0.22, *p* < 0.001; *β* = 0.11, *p* < 0.05).

**Figure 2:**
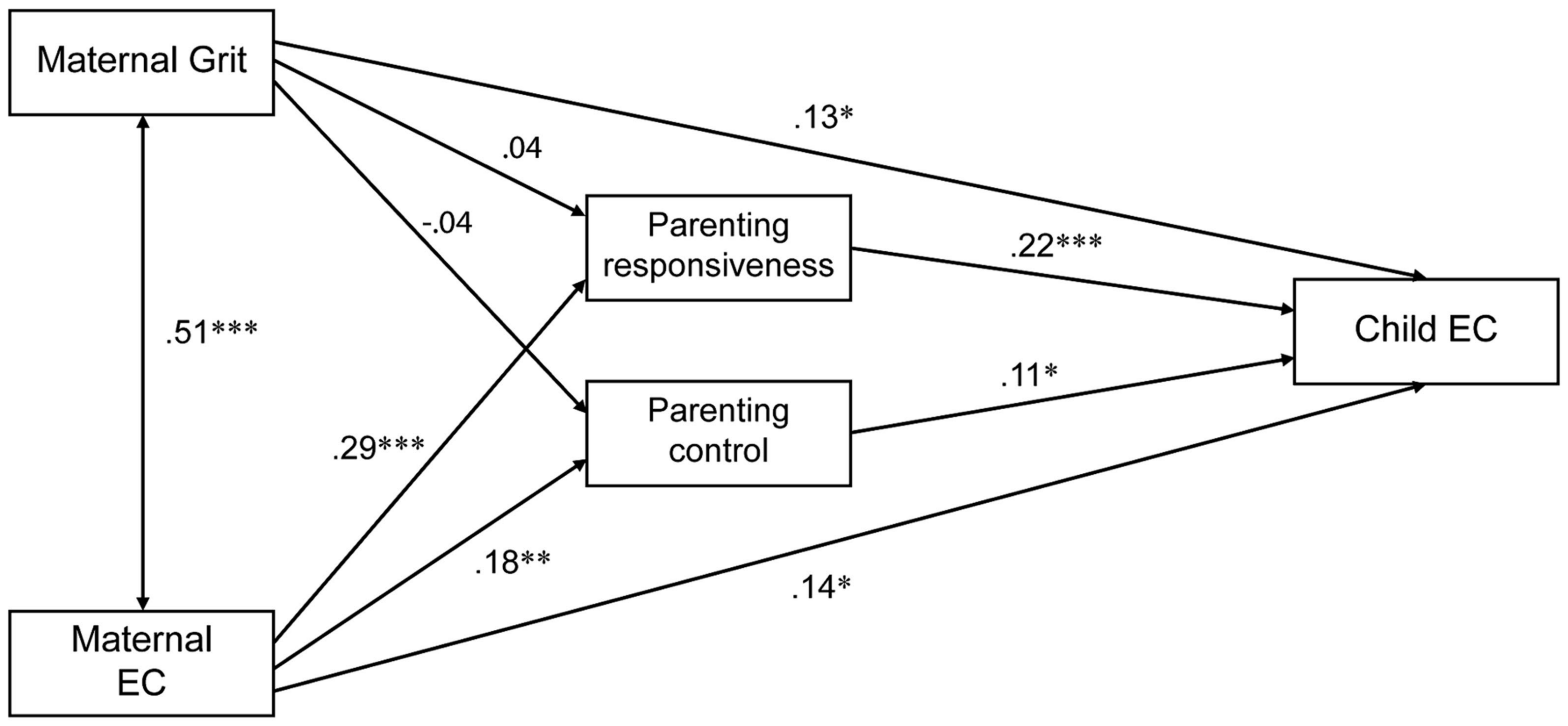
Parenting as a partial mediator in the relationship between maternal grit, maternal effortful control and child effortful control. Demographic covariates not shown. Standardized effects were reported. *p < 0.05; **p < 0.01; ***p < 0.001.

The model also did not show a high degree of fit (*χ*^2^/*df* = 90.285, *p* = 0.00, CFI = 0.728, RMSEA = 0.466), to ascertain the significance of the effects, a bootstrap nonparametric resampling procedure with 2,000 bootstrap sample simulations. Estimates of the indirect effect along with bias-corrected 95% confidence intervals (CI) were provided. Bootstrap analysis of the indirect effect of parenting on the association between maternal and child EC suggested a bias-corrected 95% CI that did not include zero (CI [0.04, 0.12]). Figure 2 illustrates the second mediation model with standardized coefficients. Children’s age and sex, SES were not statistically significant associated with child EC and were removed from subsequent mediation analysis in SEM.

## 4 Discussion

This study aimed to clarify whether maternal grit, through parenting, influences EC in early development of children, and whether maternal grit has a unique influence on child EC that is different from maternal EC. Therefore, we administered a mother-rated questionnaire focusing on toddlers (18–21 months old) in whom the earliest developmental stages of EC could be measured (using the ECBQ; Putnam et al., 2006). Additionally, mothers were asked to answer questionnaires regarding grit, EC, and parenting style. Maternal grit was positively associated with child EC, and this relationship was mediated by responsive parenting. Additionally, in models that included maternal EC, only the direct effect of maternal grit on child EC remained and the indirect effect through parenting disappeared. Furthermore, maternal EC showed not only a direct association with child EC but also an indirect association through parenting.

Our findings suggest that parenting is associated with maternal grit and child EC, and mediates their relationship. Similar to previous research, maternal responsive parenting was moderately correlated with maternal grit and child EC (Imafuku et al., 2021). Our regression analysis confirmed the relationship between maternal grit and parenting style (responsiveness/controlled) in child EC. Furthermore, to examine the role of maternal parenting, a mediation analysis revealed that maternal responsive parenting significantly mediated the relationship between maternal grit and child EC.

These results are similar to those of previous studies wherein children whose parents provided warm and supportive parenting were associated with better EC (Kochanska et al., 2000; Eisenberg et al., 2005). However, to our knowledge, the results of current study are the first to not only associate parenting with maternal grit and child EC (Imafuku et al., 2021), but also show that maternal grit mediates responsive parenting and is associated with child EC. In other words, mothers with high grit are more likely to show responsive parenting, which may contribute to fostering their children’s effortful behavior. Furthermore, regression analysis showed that the direct effect of maternal grit on child EC remained significant even after parenting and maternal EC were considered as parameters. These results indicate that parental behavior may influence child EC in ways that are not directly related to parenting. Recent studies have shown that observing persistent effortful behavior can imitatively influence infant behavior and attention, albeit in the short term (Leonard et al., 2017; Shinya and Ishibashi, 2022). Thus, the findings of the current study suggest that children may acquire effortful behavior not only through direct social interactions like parenting, but also through observation of parents’ persistent effortful attention and behaviors.

Although the relationship between maternal grit and responsive parenting weakened, considering the variance in maternal EC, maternal grit still showed a positive relationship with child EC. We found that maternal EC was not only directly associated with child’s EC but also indirectly, mediated by parenting. Previous studies have also shown that mothers with high EC provide positive parenting (Davenport et al., 2011), and that such mothers’ responses have been shown to contribute to enhancing their children’s cognitive skills, including EC (Whipple et al., 2011). To summarize, mothers who control their own behavior and show consistent parenting may foster EC in their children.

Interestingly, our results indicate that maternal grit and EC may differently affect child EC. As mentioned above, maternal grit was directly related to child EC without considering parenting as an mediating factor, whereas maternal EC was not only directly related to child EC, but also indirectly related to it via parenting. To our knowledge, this is the first study to empirically report that maternal grit and self-control (i.e., EC), which follow different processes, affect child EC in child rearing situations. Our findings also support previous research showing that grit has a different goal (having a single challenging overarching goal and working hard towards it) and timescale (longer duration) than self-control (Duckworth and Gross, 2014). However, some argue that grit and EC are similar concepts (Takahashi et al., 2021; Kevenaar et al., 2023), and that the relationship between them requires further discussion. Given the findings of the current study, at least in the context of parenting, it is possible that maternal grit and EC have different relationships with child EC. One possible explanation for this difference is the uniqueness of the sub-concepts of grit. While one of the two sub-concepts that comprise grit, persistence of effort, is relatively similar to EC, the other concept, consistency in passionate interests, is the driving force that leads to responses that actively seek and invent viable alternatives to seemingly unattainable goals and actions (Duckworth and Gross, 2014). The nature of such grit may have indirect effects from parent to child, such as imitative learning, that go beyond direct parent–child interactions in daily life. Therefore, further research on the effects of grit and EC through parenting must be conducted, considering the structural differences between the two concepts.

Future research should consider the limitations of the current research. First, although the present study showed that maternal grit and EC are directly associated with child EC, we did not examine whether above three factors were due to observational and learning effects or genetic factors. Parent and child self-regulation studies have shown the possibility of direct genetic effects (Bridgett et al., 2018). Twin studies in parental and child EC have shown a heritability of approximately 60%; although this heritability varies widely among informants, heritability estimates based on parental reports were stronger than self-reports or observations (Willems et al., 2019). As grit is a relatively new concept that has only recently received attention, few studies have focused on the possibility that parent al and child grit are directly related. Therefore, further research is needed on the genetic influence of parental and child grit. Moreover, based on the findings of this study, it would be valuable to examine not only the relationship between parental and child EC, but also the influence of parenting on child grit. Therefore, it is necessary to conduct surveys or experiments that focus on the differences between grit and EC in children. Few studies have examined whether toddlers are capable of sustained action toward long-term goals. One study measured grit in children using the Grit-S, which was assessed by their parents (Imafuku et al., 2021). In addition, maternal reports may be prone to bias, potentially resulting in less valid responses compared with an experimental assessment of children’s EC. Therefore, it is necessary to develop an age-appropriate scale that can objectively and quantitatively measure grit in children. The development of this scale will provide further insight into the differences between child grit, EC, and their developmental processes (differentiation and individual). Although we determined the children’s age range to be 18–21 months to examine precursors of grit in children, children’s EC becomes more consistent around the age of 3–4 years (Kochanska et al., 2000). Therefore, it may be difficult to generalize our results to a larger age range, it may be difficult to generalize our results to a larger age range. Thus, conducting longitudinal studies would enable the establishment of causal relationships between parenting and children’s EC.

The current study’s findings indicate that maternal grit, EC, and parenting style are associated with child EC at 18–21 months of age. Maternal grit was found to be associated with child EC via responsive parenting. Moreover, a direct association between maternal grit and child EC was observed after controlling for maternal EC. Our study provides initial evidence that maternal grit is associated with EC in young children, in a way distinct from maternal EC in child rearing situations.

## Data availability statement

The raw data supporting the conclusions of this article will be made available by the authors, without undue reservation.

## Ethics statement

The studies involving humans were approved by The Ethics Committee of University of Tokyo. The studies were conducted in accordance with the local legislation and institutional requirements. The participants provided their written informed consent to participate in this study.

## Author contributions

AJ: Conceptualization, Formal analysis, Writing – original draft, Writing – review & editing, Project administration, Visualization. MI: Conceptualization, Data curation, Funding acquisition, Investigation, Methodology, Project administration, Writing – original draft, Writing – review & editing. YS: Conceptualization, Funding acquisition, Investigation, Methodology, Project administration, Writing – original draft, Writing – review & editing. SI: Conceptualization, Funding acquisition, Supervision, Writing – review & editing.
